# Supplementary material for: Achilles tendon thickness and serum asprosin level significantly increases in patients with polycystic ovary syndrome
Source: PeerJ. 2024 Aug 22;12:e17905. doi: 10.7717/peerj.17905 (PMC11345002; doi:10.7717/peerj.17905)
Supplement: Supplemental Information 2 [file peerj-12-17905-s002.docx]

STROBE Statement—checklist of items that should be included in reports of observational studies

|  | **Item No.** | **Recommendation** | **Page No.** | **Relevant text from manuscript** |
| --- | --- | --- | --- | --- |
| **Title and abstract** | 1 | (*a*) Indicate the study’s design with a commonly used term in the title or the abstract | 4 | Line 47 |
|  |  | (*b*) Provide in the abstract an informative and balanced summary of what was done and what was found | 5 | Line 63-67 |
| **Introduction** |  |  |  |  |
| Background/rationale | 2 | Explain the scientific background and rationale for the investigation being reported | 6 | Line 88-91 |
| Objectives | 3 | State specific objectives, including any prespecified hypotheses | 6 | Line 93-95 |
| **Methods** |  |  |  |  |
| Study design | 4 | Present key elements of study design early in the paper | 6 | Line 99-100 |
| Setting | 5 | Describe the setting, locations, and relevant dates, including periods of recruitment, exposure, follow-up, and data collection | 6 | Line 99-100 and Line 109-113 |
| Participants | 6 | (*a*) *Cohort study*—Give the eligibility criteria, and the sources and methods of selection of participants. Describe methods of follow-up  *Case-control study*—Give the eligibility criteria, and the sources and methods of case ascertainment and control selection. Give the rationale for the choice of cases and controls *Cross-sectional study*—Give the eligibility criteria, and the sources and methods of selection of  participants | 6 | Line 101-108 |
|  |  | (*b*) *Cohort study*—For matched studies, give matching criteria and number of exposed and unexposed  *Case-control study*—For matched studies, give matching criteria and the number of controls per  case | NA | NA |
| Variables | 7 | Clearly define all outcomes, exposures, predictors, potential confounders, and effect modifiers.  Give diagnostic criteria, if applicable | 6 | Line 100-108 |
| Data sources/ measurement | 8* | For each variable of interest, give sources of data and details of methods of assessment (measurement). Describe comparability of assessment methods if there is more than one group | 6-7 | Line 113-140 |
| Bias | 9 | Describe any efforts to address potential sources of bias | 6 | Line 108-109 and Line 111-113 |
| Study size | 10 | Explain how the study size was arrived at | 6 | Line 99-100 |

Continued on next page

| Quantitative variables | 11 | Explain how quantitative variables were handled in the analyses. If applicable, describe which groupings were chosen and why Page no 6-7 Line 113-129 |
| --- | --- | --- |
| Statistical methods | 12 | (*a*) Describe all statistical methods, including those used to control for confounding Page No 7-8 Line 142-151 |
|  |  | (*b*) Describe any methods used to examine subgroups and interactions Page No 7-8 |
|  |  | (*c*) Explain how missing data were addressed NA |
|  |  | (*d*) *Cohort study*—If applicable, explain how loss to follow-up was addressed NA  *Case-control study*—If applicable, explain how matching of cases and controls was addressed NA  *Cross-sectional study*—If applicable, describe analytical methods taking account of sampling strategy NA |
|  |  | (*e*) Describe any sensitivity analyses NA |
| **Results** |  |  |
| Participants | 13* | 1. Report numbers of individuals at each stage of study—eg numbers potentially eligible, examined for eligibility, confirmed eligible, included in the study, completing follow-up, and analysed   Page no 16 Table 1 |
|  |  | (b) Give reasons for non-participation at each stage NA |
|  |  | (c) Consider use of a flow diagram NA |
| Descriptive data | 14* | (a) Give characteristics of study participants (eg demographic, clinical, social) and information on exposures and potential confounders Page no 16 Table 1 |
|  |  | (b) Indicate number of participants with missing data for each variable of interest NA |
|  |  | (c) *Cohort study*—Summarise follow-up time (eg, average and total amount) NA |
| Outcome data | 15* | *Cohort study*—Report numbers of outcome events or summary measures over time NA |
|  |  | *Case-control study—*Report numbers in each exposure category, or summary measures of exposure NA |
|  |  | *Cross-sectional study—*Report numbers of outcome events or summary measures 8 Line 156-165 |
| Main results | 16 | 1. Give unadjusted estimates and, if applicable, confounder-adjusted estimates and their precision (eg, 95% confidence interval). Make clear which confounders were adjusted for and why they were included Page no 8 Line 154-165 |
|  |  | (*b*) Report category boundaries when continuous variables were categorized NA |
|  |  | (*c*) If relevant, consider translating estimates of relative risk into absolute risk for a meaningful time NA  Period |

Continued on next page

| Other analyses | 17 | Report other analyses done—eg analyses of subgroups and interactions, and sensitivity analyses NA |
| --- | --- | --- |
| **Discussion** |  |  |
| Key results | 18 | Summarise key results with reference to study objectives 8 Line 168-172 |
| Limitations | 19 | Discuss limitations of the study, taking into account sources of potential bias or imprecision. Discuss both direction and magnitude of any potential bias Page no 11 Line 233-237 |
| Interpretation | 20 | Give a cautious overall interpretation of results considering objectives, limitations, multiplicity of 9-10 Line 179-232  analyses, results from similar studies, and other relevant evidence |
| Generalisability | 21 | Discuss the generalisability (external validity) of the study results 11 Line 240-244 |
| **Other information** | | |
| Funding | 22 | Give the source of funding and the role of the funders for the present study and, if applicable, for the original study on which the present article is based Page no 3 Line 19-20 |

*Give information separately for cases and controls in case-control studies and, if applicable, for exposed and unexposed groups in cohort and cross-sectional studies.

**Note:** An Explanation and Elaboration article discusses each checklist item and gives methodological background and published examples of transparent reporting. The STROBE checklist is best used in conjunction with this article (freely available on the Web sites of PLoS Medicine at [http://www.plosmedicine.org/,](http://www.plosmedicine.org/) Annals of Internal Medicine at [http://www.annals.org/,](http://www.annals.org/) and Epidemiology at [http://www.epidem.com/).](http://www.epidem.com/)) Information on the STROBE Initiative is available at [www.strobe-statement.org.](http://www.strobe-statement.org/)
